# Uptake Difference by Somatostatin Receptors in a Patient with Neuroendocrine Tumor: 99mTc-Octreotide Uptake in the Lung without Uptake in Liver Lesions

**DOI:** 10.4274/mirt.02996

**Published:** 2015-11-02

**Authors:** Elahe Pirayesh, Mahasti Amoui, Majid Assadi

**Affiliations:** 1 Shahid Beheshti University of Medical Sciences, Shohada-e Tajrish Medical Center, Department of Nuclear Medicine, Tehran, Iran; 2 Bushehr University of Medical Sciences, The Persian Gulf Nuclear Medicine Research Center, Bushehr, Iran

**Keywords:** Radionuclide imaging, neuroendocrine tumors, 99mTc-octreotide, receptors, somatostatin

## Abstract

The diagnostic value of somatostatin receptor scintigraphy (SRS) in detecting tumors has been assessed in a number of studies. We present a 30-year-old female with a history of eight months cough and left shoulder pain. Radiologic evaluation showed pulmonary mass and hepatic lesions, which were pathologically diagnosed as neuroendocrine carcinoma. 99mTc-octreotide scan demonstrated that the pulmonary lesion was positive for somatostatin receptor (SSTR), while the liver metastases were SSTR negative. The present case highlights the significance of a differential uptake pattern by somatostatin receptors in SRS in patients with neuroendocrine tumors.

## Abstract (tr)

Somatostatin reseptör sintigrafisinin (SRS) tümör saptamadaki tanısal değeri bir dizi çalışmada değerlendirilmiştir. Bu yazıda sekiz aylık öksürük ve sol omuz ağrısı öyküsü olan 30 yaşındaki bir kadın hasta sunulmaktadır. Radyolojik değerlendirme ile akciğerde kitle ve karaciğer lezyonları saptanan hastada patolojik olarak nöroendokrin karsinom saptandı. 99mTc-oktreotid taramasında akciğer lezyonu somatostatin reseptörü (SSTR) için pozitif iken karaciğer metastazları SSTR negatif idi. Bu olgu nöroendokrin tümörlerde SRS ile somatostatin reseptörlerinin farklı tutulum göstermesinin önemini vurgulamaktadır.

## INTRODUCTION

Somatostatin receptor scintigraphy (SRS) is a functional imaging modality that is used to evaluate neuroendocrine tumors (1). The diagnostic value of SRS in detecting tumors has been assessed in a number of studies (1). Its uptake has been shown in different cell lines such as lymphocytes, fibroblasts, and endothelium (2). Herein, we present a 30-year-old female with neuroendocrine tumor of the lung and liver, with pulmonary 99mTc-octreotide uptake on SRS.

## CASE REPORT

**Figure 1:**
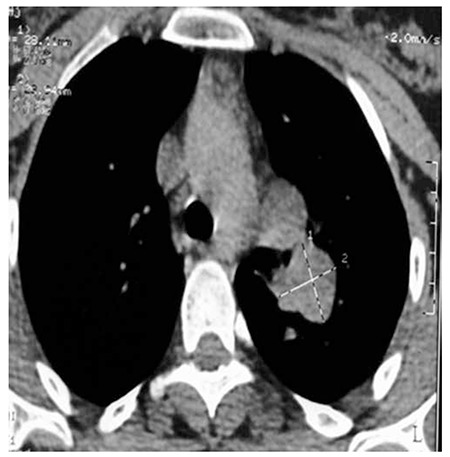
Computed tomography scan showing a large mass in the left lung field

**Figure 2:**
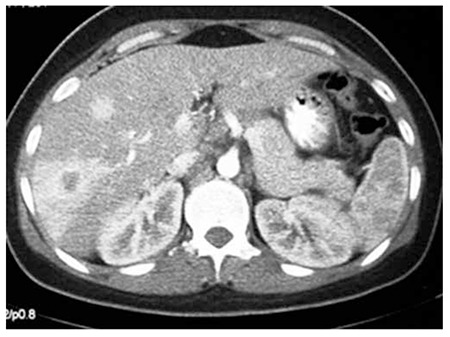
Computed tomography scan showing multiple hypodense regions in the right liver lobe

A 30-year-old female, who had been suffering from a non-productive cough for 8 months and left shoulder pain, was found to have a large mass in the left lung (Figure 1). Further evaluation by abdominal sonography and computed tomography (CT) scan revealed multiple hepatic lesions (Figure 2). A CT guided biopsy of the liver lesions was performed. The liver sections showed poorly cohesive nests of epithelial cells with plasmocytoid and signet ring morphology, and solitary infiltrating cells with vascular permeation in a non-cirrhotic liver parenchyma, suggesting metastatic undifferentiated carcinoma with signet ring feature.

The immunohistochemistry (IHC) results were positive for EMA, CK, Chromogranin, Ki67 (1%) indices, but were negative for TTF1, GCDFP15, Heppar, Ck7, and Ck20 indices. IHC findings were in favor of metastatic low-grade neuroendocrine carcinoma.

**Figure 3:**
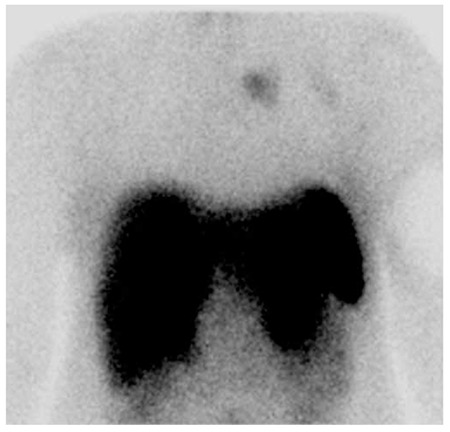
Technetium-99m-labeled octreotide acetate scintigraphy in the planar view of the thorax (anterior). This was performed 15 minutes after injection of 740 MBq technetium-99m-labeled octreotide acetate. There was an area of abnormal uptake in the left lung field

**Figure 4:**
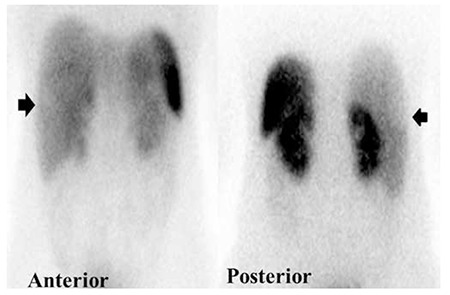
Technetium-99m-labeled octreotide acetate scintigraphy in the planar view of the abdomen (anterior and posterior). This was performed 3 hours after injection of 740 MBq technetium-99m-labeled octreotide acetate. There was a suspicious photopenic area in the posterior view of the right lobe of liver

**Figure 5:**
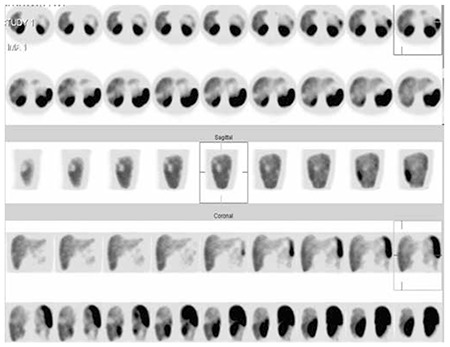
Technetium-99m-labeled octreotide acetate scintigraphy in the SPECT mode. It revealed some photopenic regions in the liver, compatible with the hepatic masses on computed tomography images

She was healthy with an unremarkable past medical history, and she was not on any medications. Scintigraphic imaging was done 15 minutes and 3 hours after IV injection of 740 MBq (20 m mCi) 99mTc-Edda-tricine-Hynic-Tyro-octreotide, and an increased radiotracer uptake in the lung mass was identified (Figure 3). On planar images, there was a suspicious photopenic area in the posterior view of the liver (Figure 4). A SPECT was done and revealed some photopenic regions in the liver, compatible with the hepatic masses on CT images (Figure 5).

## LITERATURE REVIEW AND DISCUSSION

Molecular imaging alters the diagnosis and treatment of patients with neuroendocrine tumors. SSTR scintigraphy has become the method of choice for functional imaging of these tumors (3). SRS with [111In-DTPA0] octreotide has established its role in the diagnosis and staging of gastroenteropancreatic neuroendocrine tumors (GEP-NETs) (4). In addition, radiolabelled metaiodobenzylguanidine (MIBG) has been applied for many years to detect carcinoid tumors (5). Somatostatin analogues have been labelled with different positron-emitting isotopes, such as Gallium-68 (68Ga) and Copper-64 (64Cu) (6). Furthermore, other PET radiotracers such as 18F-dihydroxy-phenyl-alanine (18F-DOPA) and 11C-labelled 5-hydroxytryptophan (11C-5-HTP) were introduced with promising results in detecting GEP-NETs (7).

Somatostatin receptors are overexpressed at the cell membrane and peritumoral vessels of a large variety of NETs. Although, various SSTR subtypes are expressed in tumors, SSTR2 is the predominant one, and it provides the molecular basis for clinical application of SS analogues for diagnostic and therapeutic purposes (8). However, it is well known that tumors frequently acquire cellular heterogeneity (9). This is related to tumorigenesis that is not a static entity: the tumor initiates from a genetically normal cell and proliferates into billions of malignant cells, during which it accumulates many mutations (9). There is strong evidence for the co-existence of genetically divergent tumor cell clones within a variety of tumors, and it has gained attention especially regarding response to therapy (9). It could also be a potential factor for sampling error (10). An IHC investigation reported heterogeneity in SSTR subtype expression between primary vs. metastatic NETs, as well as among hepatic metastases (11). There is also evidence for heterogeneity of Ki67 index in metastatic NETs (11).

In endocrine tumors, the presence of SSTRs are associated with well differentiation, low grade tumor and good response to somatostatin analog (octreotide) treatment (12,13).

While most well-differentiated endocrine tumors and islet cell carcinomas are SSTR-positive, and therefore responsive to somatostatin analog therapy; the poorly differentiated endocrine tumors are generally SSTR-negative and hardly benefit from somatostatin analog therapy (12,13,14,15).

The present case shows an uptake difference by somatostatin receptors in somatostatin receptor scintigraphy of a NET in the lung and its liver metastases, which can be explained by tumor heterogeneity and a different pattern of SSTR expression. In addition, it highlights the importance of SPECT study in detecting photopenic regions. Nuclear physicians should also be aware that all metastases do not necessarily appear as hot lesions. Photopenic areas require special emphasis.
